# Novel Strategies for Immunotherapy in Multiple Myeloma: Previous Experience and Future Directions

**DOI:** 10.1155/2012/753407

**Published:** 2012-05-10

**Authors:** Ivetta Danylesko, Katia Beider, Avichai Shimoni, Arnon Nagler

**Affiliations:** ^1^Division of Hematology and Bone Marrow Transplantation, Chaim Sheba Medical Center, Tel Aviv University, Tel Hashomer, Israel; ^2^Cord Blood Bank, Chaim Sheba Medical Center, Tel Aviv University, Tel Hashomer, Israel

## Abstract

Multiple myeloma (MM) is a life-threatening haematological malignancy for which standard therapy is inadequate. Autologous stem cell transplantation is a relatively effective treatment, but residual malignant sites may cause relapse. Allogeneic transplantation may result in durable responses due to antitumour immunity mediated by donor lymphocytes. However, morbidity and mortality related to graft-versus-host disease remain a challenge. Recent advances in understanding the interaction between the immune system of the patient and the malignant cells are influencing the design of clinically more efficient study protocols for MM. Cellular immunotherapy using specific antigen-presenting cells (APCs), to overcome aspects of immune incompetence in MM patients, has received great attention, and numerous clinical trials have evaluated the potential for dendritic cell (DC) vaccines as a novel immunotherapeutic approach. This paper will summarize the data investigating aspects of immunity concerning MM, immunotherapy for patients with MM, and strategies, on the way, to target the plasma cell more selectively. We also include the MM antigens and their specific antibodies that are of potential use for MM humoral immunotherapy, because they have demonstrated the most promising preclinical results.

## 1. Introduction

In spite of recent advances [1, 2], MM remains an incurable disease, and new approaches that induce long-term tumor regression and improve disease outcome are needed.

 Autologous stem cell transplantation is a common treatment for MM and results in effective cytoreduction. However, the curative outcome remains elusive due to chemotherapy-resistant disease [3]. A promising route to overcome chemotherapy resistance is the development of immunotherapeutic approaches that target and eliminate myeloma cells more selectively.

 A critical indication that immunotherapy is effective is that tumor-associated antigens (TAAs) are expressed in the tumor cells if disease reemerges after therapy. Vaccination strategies targeting single antigens and whole-cell approaches have shown promise in clinical studies.

They also have the advantage of presenting patient-specific and potentially unidentified antigens to immune effector cells.

 Monoclonal antibodies (mAbs) have been evaluated in preclinical and clinical studies. Potential mAb candidates include growth factors and their receptors, other signalling molecules, and antigens expressed exclusively or predominantly on MM cells. Therapy with mAb may involve a range of mechanisms, including antibody-dependent cellular cytotoxicity (ADCC), complement-dependent cytotoxicity (CDC), interference with receptor-ligand interactions, and mAb conjugation to radioisotopes or toxins [4].

Effector cell dysfunction and the increased number of regulatory T cells in patients with malignancy may limit the efficacy of immunotherapeutic approaches. Strategies to improve immunotherapy for MM involve the depletion of T regulatory cells, combining active and passive immunotherapy, the use of cytokine adjuvants, and using immunotherapy in conjunction with autologous and allogeneic transplantation.

 The unique value of immunotherapy, in allogeneic transplantation, is the graft-versus-disease effect mediated by alloreactive lymphocytes, which attack the tumor.

However, the significant morbidity and mortality due to regimen-related toxicity and graft-versus-host disease (GvHD) pertain [5].

Immunotherapy is promising area of investigation that focuses on developing strategies to elicit myeloma-specific immune responses to eliminate the malignant plasma cell selectively.

## 2. Tumor-Specific Immunity and Immune Evasion: The Role of the Adoptive and Innate Immune System in Controlling MM

MM is associated with a variety of immune defects; therefore, immunotherapy is particularly challenging. It is considered, at least to a certain extent, to be controlled by the adaptive immune system. This hypothesis is supported by the fact that the therapeutic effect of alloSCT is mediated in part by immune effects exerted by donor-derived T cells and that donor T cells infused into MM patients are capable of inducing remission in case of relapse [6, 7].

 The development of effective tumor-specific immunotherapy requires addressing several basic issues concerning tumor cell biology and the complex interaction between cancer cells and host immunity.

Tumor cells may evade host immunity through a variety of mechanisms. Some may contribute to myeloma cell ‘‘tolerance,” including myeloma-derived cytokines such as transforming growth factor-b (TGF-b), which suppresses B cells and T cells via inhibition of interleukin-2 (IL-2) autocrine pathways, inadequate antigen presentation, resistance to NK cell lysis, and defective T, B, and NK cells [8]. Much data suggests that early-stage cancers are eliminated by immune surveillance, whereas established tumors are more likely to induce immune tolerance [9].

Tumor-specific CD4+ T cells have a central function in the immune response against cancer [10, 11]. Early studies in rats and mice indicated that adoptive transfer of tumour-specific CD4+ T cells may be very efficient in eradicating established cancers [12, 13]. CD4+ T cells are required for activation of tumour-specific cytotoxic CD8+ T cells [14], but they can also eradicate cancer in the absence of CD8+ T cells [15, 16]. Tumor-specific CD4+ T cells recognize antigenic peptides presented by MHC class II molecules. However, most cancer cells are MHC class II negative and therefore cannot be directly recognized by CD4+ T cells. Tumor-specific CD4+ T cells overcome this obstacle by collaborating with macrophages and dendritic cells [17]. These professional antigen-presenting cells endocytose TSA, process it, and display antigenic peptides on their MHC class II molecules for recognition by tumor-specific CD4+ T cells [10, 18, 19]. The number and function of T cell subsets were reported to be abnormal in patients with MM. The CD4 : CD8 ratio inverted, and the Th1 : Th2 ratio among CD4+ cells is abnormal [20]. T cells from MM patients were shown to function aberrantly [21]. In addition, the levels of expression of CD28 and cytotoxic T lymphocyte-4 (CTLA-4) costimulatory molecules required for T cell activation and inhibition, respectively, were downregulated in T cells derived from MM patients [22]. Tumor cells express a variety of factors that suppress the function and development of APCs and T cells. The B7 family of cosignaling molecules is expressed on the surface of T lymphocytes and is crucial for their optimal activation, as well as for the prevention of immunologic tolerance. These cosignaling molecules not only provide critical positive signals that stimulate T cell growth, upregulate cytokine production, and promote T cell differentiation but also contribute key negative signals that limit, terminate, and/or attenuate T cell responses [23, 24].

 Although the antibodies may trigger direct antitumor activity through their Fab2 portion causing apoptosis of tumor cells [25], they more often mediate damage of cancer cells by recruiting other effector systems of innate immunity through their Fc portion. Such effector cells include mononuclear and polymorphonuclear leukocytes that phagocytise opsonized tumor cells and NK cells primarily involved in the process of ADCC [26]. Complement is the other soluble effector system of innate immunity that can be recruited by mAbs to control tumor growth CDCC [27, 28].

 The role of innate effector cells, such as macrophages, NK cells, NKT cells, and *γδ*T cells, in natural tumor immunity and tumor immunotherapy has been revisited [29, 30]. NK cells are cytotoxic lymphocytes that have the ability to lyse certain tumor and virus-infected cells, without prior immunization [31]. The cytotoxic activity of NK cells is tightly controlled by a balance between activating and inhibitory signals from receptors on the cell surface [32]. Activating receptors include the natural cytotoxicity receptors and NKG2D, all of which push the balance toward cytolytic action through engagement with separate ligands on the target cell surface [32]. The role of autologous NK cells in the immune recovery, which is a strong prognostic indicator for survival after autologous stem cell transplantation (ASCT), was highlighted by Porrata et al. [33], who showed that reinfusion of autologous NK cells correlates with absolute lymphocyte recovery after ASCT for MM and non-Hodgkin's lymphoma.

 Defective NK cells have also been noted in patients with MM [34]. This is of major importance since NK cells have antimyeloma activity [35, 36]. In the setting of AlloSCT for MM, there is emerging evidence that donor NK cells along with donor Tlymphocytes exhibit anti-MM activity [37]. In another study, it was shown that, after coincubation of NK cells from normal volunteers with myeloma cells from three different MM cell lines and fresh BM samples from nine myeloma patients, myeloma cells were susceptible to NK cell lysis, even in the absence of IL-2 [36]. Of note, CD34 hematopoietic stem cells, as well as peripheral blood mononuclear cells (PBMNCs), were completely resistant to NK cell killing under similar conditions [36]. Recently, it has been shown that autologous NK cells from myeloma patients expanded *ex vivo* with IL-2 displayed significant cytotoxic activity against primary autologous plasma cells [38]. Furthermore, it has been demonstrated that the infusion of haploidentical killer-cell immunoglobulin-like receptor (KIR) ligand-mismatched NK cells in the autologous MM setting resulted in 50% near complete remission of relapsed MM patients [39]. However, *ex vivo* or *in vivo* expansion of the NK cells with IL-2 carries a dose-limiting toxicity.

 The role of dendritic cells (DCs) is dichotomous; they may present both antigens, appropriate stimulator molecules to initiate an adaptive immune response, or they may induce tolerance and release anti-inflammatory signals. Circulating DCs from MM patients were shown to be dysfunctional because they failed to upregulate costimulatory molecules required for activation [40]. It was suggested that a reduced function of DCs indicates the progression of the disease [40]. Cytokines, such as IL-6, TGF-b, IL-10, and vascular endothelial growth factor (VEGF), which were actively produced by myeloma cells [40] and were found to be in the tumor microenvironment as well as in the serum [41], played a role in preventing the development of functional DCs. Furthermore, DCs from MM patients had reduced phagocytic capacity [42]. In addition, monocyte-derived DCs exhibited downregulated expression of activation markers and impaired presentation capacity to T cells [41]. Impaired activity of DCs may be linked to the upregulation of Tregs [43]. T cell tolerance to tumor-associated antigens plays a significant role in immune evasion by tumors [44, 45]. Naturally occurring and adaptive regulatory T cells (Tregs) are anergic cells with suppressive capabilities that constitute 5–10% of CD4 cells. These cells are induced early during tumor development and were shown to contribute to tumor tolerance [46, 47].

 The presence of Tregs in tumors is associated with a poor prognosis [48]. Patients with many different types of cancers had increased numbers of Tregs in their blood, tumor mass, and draining lymph nodes [49, 50]. Increased numbers of Tregs were found in patients with MM as well [51–53]. Therapeutic approaches for breaking tolerance to tumor cells have been tried; the depletion of Tregs is the most studied strategy [54–56]. Nevertheless, despite the tumor antigen-specific immunity [57], the tumors were not completely rejected [58]. Thus, it is essential to reveal the mechanism leading to Treg expansion for developing strategies to eliminate them and to improve the results of cancer immunotherapy [59].

 There is also emerging evidence that the cellular bone compartment affects MM cell growth and progression. This is supported by the observation that osteoclasts can support long-term survival and proliferation of primary MM cells [60, 61], and osteoblasts (OB) may impede MM cell growth [62, 63]. Thus, targeting these cellular elements may also favorably affect disease control. The BM microenvironment in MM controls the tumor growth, myeloma cell survival [64] and drug resistance [65, 66]. In turn, MM cells were suggested to modify the BM microenvironment in which they reside in several ways including induction of osteoclastogenesis and suppression of osteoblast activity, both leading to impaired bone formation [67]. BM-derived mesenchymal stromal cells (MSCs) are precursors of osteoblasts and preferentially differentiate into bone forming cells upon *in vitro *culture and *in vivo* introduction. MM cells were suggested to target MSCs thereby diverting their functions to serve the MM cells. This idea led to studies of the functions of MSCs derived from MM patients (MM-derived MSCs) compared to those of healthy individuals; it was suggested that MSCs from myeloma patients exhibit defective functions [68–70]: MM-derived MSCs were reported to exhibit decreased colony-forming unit number [70], growth impairment [70], reduced osteogenic differentiation [68] and increased IL-6 secretion [68, 70].

To summarize, the task of developing effective immunotherapy for cancer relies on the identification of appropriate tumor targets, the augmentation of antigen-presenting and effector cell function, and the reversal of the tumor-mediated immunosuppressive state [71]. In this review we focus on MM antigens and their specific antibodies, which have demonstrated the most promising results in preclinical studies and are therefore the best candidate for future MM humoral immunotherapy.

## 3. Myeloma-Specific Antigens and Vaccines: Idiotype—Preclinical Studies

 The myeloma-specific antigen that can be targeted by immunotherapy is the idiotype (Id) protein representing the variable segment of the monoclonal immunoglobulin generated in the plasma cell clone [72]. Targeting of the idiotype protein by humoral or cellular immune mechanisms, in preclinical models, results in death of the tumor cells and disease regression. Induction of protective antitumor immunity through immunization with a myeloma idiotype has been most extensively studied with the murine plasmacytoma MOPC-315 model. In this model, it was shown that weekly immunizations with tumor-derived paraprotein protect syngeneic mice against a subsequent challenge with MOPC-315 cells [73]. Idiotype-specific T cells at a low frequency were detected in 90% of patients with MM or MGUS [74, 75]. In addition, transfer of idiotype-specific CD4+ T cells has been shown to be protective against tumor challenge [76]. Antigen-specific responses, of both CD4 and CD8 positive T cells, upon *in vitro *stimulation with autologous paraprotein have been described in patients with monoclonal gammopathies [74]. Induction of cytotoxic T cell activity against autologous myeloma cells was also shown for stimulation with idiotype-loaded dendritic cells [77, 78]. Consistent with these results, several authors have shown that T cells in myeloma patients respond to peptides corresponding to complementarity-determining region I–III of heavy and light chains of the autologous M-component [79, 80]. Yi found [81] that idiotype-induced T cell stimulation was mainly confined to the CD4+ subset in most of the patients examined and was MHC class II restricted. Idiotype-specific CD8+ T cells were also demonstrated, but at a lower frequency. Idiotype-specific CD4+ and CD8+ T cells were mainly of the type 1 subsets, as judged by their secretion of interferon (IFN)-*γ* and interleukin (IL)-2 [82, 83]. Moreover, the number of individuals who had an idiotype-specific response of the T-helper-1 (Th1) type (IFN-*γ*- and/or IL-2-secreting cells) [84] was significantly higher for patients with indolent disease (MGUS and MM stage I) than for those with advanced MM (stage II/III). In contrast, cells secreting the Th2-subtype cytokine profile (IL-4 only) [84] were observed more frequently in patients with advanced MM (stage II/III) [75].

Collectively, these findings indicate that the existing idiotype-specific immune response is too weak to control the growth of myeloma cells *in vivo*. It is possible that a shift from an idiotype-specific type-1 response, that is, Th1 and T cytotoxic-1 (Tc1) [85], in early MM to a type 2 response (Th2 and probably Tc2) [85] in advanced disease occurs. These studies provide indirect evidence that idiotype-specific T cells may have a regulatory impact on human tumor B cells.

## 4. Clinical Trials of Idiotype Vaccination for MM

Native idiotype protein can be obtained from the serum of myeloma patients, making vaccination trials relatively easy. Injection of paraprotein alone may lead to an increased cellular and humoral immune responses, but it seems insufficient to generate sustained antimyeloma immunity [86]. Intradermal injections of paraprotein, combined with subcutaneous administration of GM-CSF at the same site, induced an increase in the numbers of IFN*γ*- and IL-2-secreting T cells [87]. This response was present in CD4+ and CD8+ T cell subsets and could be inhibited by blockade of MHC class I molecules. Furthermore, production of idiotype-specific IgM was induced *in vivo*. However, there was no clear indication of clinical efficacy since the paraprotein levels remained essentially unchanged, and DTH (delayed-type hypersensitivity reaction) responses to idiotype protein were not detected.

 In contrast, in subcutaneous vaccination with keyhole limpet haemocyanin (KLH)-coupled paraprotein and additional adjacent injections of GM-CSF in patients after a high-dose chemotherapy and ASCT, DTH reactions to the vaccine were induced in 85% of patients, but *in vitro *testing provided little evidence for specific T cell immune responses [88, 89].

 A potential concern with the use of idiotype-based vaccination approaches in MM is that plasma cells only express the idiotype protein weakly, and idiotype may be associated with the development of tolerance. One strategy for targeting myeloma by host effector cells is the genetic manipulation of T cells such that the idiotype antibody is expressed and induces signalling via the T cell receptor.

 When patients in stage I disease were immunized with idiotype in conjunction with IL-12 +/− GM-CSF, there was a decrease in circulating clonal cells as detected by quantitative PCR monitoring in four of six patients [90]. Finally, intradermal immunization with uncoupled recombinant idiotype in conjunction with GM-CSF induced idiotype-specific T cell reactivity in a patient with advanced myeloma [91].

 Idiotype-loaded dendritic cells (DCs) have been used by various groups as vaccines in MM patients, mostly in the setting of clinical remission after autoSCT [92–96]. Although the patient characteristics and vaccine particularities preclude firm comparisons between these trials, they nevertheless have collectively shown that the induction of cellular immune responses is possible in the setting of minimal disease burden after ASCT. However, no real evidence has been obtained in these Phase I and II trials that the natural course of the disease has been altered by idiotype vaccination, and efforts to improve the immunogenicity of the vaccination are ongoing.

## 5. Myeloma-Specific Tumor Antigens

 A variety of tumor-associated antigens have been identified in myeloma cells that may be targeted selectively by the immunity of the host. These include the cancer testis (CT) antigens, MUC1, HM1.24, CYP1B1, SP17, PRAME, Wilms' tumour 1 (WT1), and heat shock protein gp96 [97–102].

 MUC1 is a physiologically highly glycosylated epithelial mucin. Since the molecule is often expressed but severely underglycosylated on malignant cells, it may be recognized by cytotoxic T cell lymphocyte (CTL) toxicity in a MHC-unrestricted manner [103]. This effect has also been shown in myeloma [104]. Furthermore, HLA class-I-restricted peptide target epitopes have also been identified within the MUC1 sequence, and the majority of myelomas appear to express and present these epitopes to T cells [97].

 WT1 is a zinc finger transcription factor with overexpression in myeloid malignancies [105, 106]. While WT1 is also frequently expressed, albeit at lower level, in lymphoid malignancies, myeloma cells may be efficiently recognized and lysed by WT1-specific CTL [107].

 CD317/HM1.24, a cell surface protein involved in cell signaling [108], is another potential tumor-associated antigen overexpressed in MM [109]. HM1.24-specific CTL can be induced in healthy volunteers and MM patients [110].

 Recently, it has been shown that the pituitary tumor transforming gene 1 is expressed aberrantly in multiple myeloma and may serve as a target for cellular immunotherapy [111].

 The RHAMM is an immunogenic antigen that is strongly expressed in several hematologic malignancies [112, 113] and induces humoral and cellular immune responses [114–119].

 CT antigens represent a family of proteins that are expressed in a variety of tumors, while their presence in normal tissue is limited to the testis and placenta. Several groups have described that CT antigens are also expressed by myeloma cells [98, 120–125].

 CT antigens are commonly capable of inducing antibody-mediated and T cell-mediated immunity in MM patients [100]. CT Ag-specific T cells can be detected in the blood of myeloma patients and appear to be functionally competent [124, 126]. Depending on the patient population and the method used to detect CT gene expression, there appears to be a trend towards higher likelihood of expression with advanced stage [121, 123] and presence of cytogenetic abnormalities [124], both representing adverse prognostic factors in myeloma. These antigens represent potential markers for minimal residual disease (MRD) after ASCT and could also be used to target myeloma cells remaining in the patients' BM after standard therapy. In addition, in MM, expression of CT antigens has been shown to be strongly correlated to the clinical outcome; that is, the presence of CT antigens expression has been linked to shorter survival [127].

 Baseline expression frequencies, measured by RT-PCR, determined MAGE-C1/CT7 as the most frequently detected antigen, possibly perform a gatekeeper function for the other antigens examined. Importantly, 80% of the patients with a significant number of plasma cells expressed at least one of these antigens investigated [128].

 A novel CT antigen, ropporin, is a testis-specific protein localized in the sperm flagella. Comparing ropporin expression in healthy and MM samples, ropporin expression positive signals were found in 44% of the MM primary samples. The immunogenicity of ropporin was confirmed by the presence of specific antibodies detected by enzyme-linked immunosorbent assay in patients' serum [129].

 The gene expression of CT antigen in relapse samples and in newly diagnosed MM cases was evaluated [130, 131]. The CT antigen expression after treatment was shown for a limited number of CT antigens including PASD1, CTAG1B, and MAGEC1/CT7 [123, 124, 128, 132, 133]. Multivariate analysis demonstrated that for the set of newly diagnosed cases shorter overall survival was associated with the presence of MAGEA6 and CDCA1, and MAGEA9 was associated with a shorter overall survival in [130]. In the set of the relapse cases, the presence of CTAG2 was associated with a shorter progression free-survival and the presence of SSX1 with shorter overall survival [130].

 NY-ESO-1 is the most immunogenic of the CT antigens [99]. NY-ESO-specific CTLs generated from patients with MM were shown to kill primary myeloma cells, normal cells pulsed with a NY-ESO-1 peptide, but not normal cells pulsed with an irrelevant peptide. Spontaneous humoral and CD8− T cell-mediated responses to NY-ESO-1 have been identified in patients with advanced disease [99, 124, 126]. Vaccination strategies targeting NY-ESO-1 may be effective at inducing specific antimyeloma immunity, and a clinical trial evaluating the efficacy of an NY-ESO peptide vaccination given in conjunction with GM-CSF is underway [99].

 Importantly, the finding that immune responses against CT antigens are induced by alloSCT [100] suggests that this class of tumor antigens might indeed represent natural targets for donor-derived alloimmune or even spontaneous antimyeloma immune responses. Interestingly, in patients undergoing an allogeneic transplantation, antibody responses to NY-ESO-1 were detected after transplantation, suggesting that this may represent a target for the graft-versus-myeloma effect. LAGE-1 mRNA was detected in 49% of MM patients [134]. Recently, de Carvalho et al. reported that LAGE-1a mRNA was more frequent than LAGE-1b expression in MM cases [135]. The LAGE-1a protein has 84% similarity with the NY-ESO-1 protein, and the authors identified seven peptides present in both CTAs that were recognized by T lymphocytes in different tumors. Because spontaneous humoral immunity against NY-ESO-1 was not detected before the allogeneic transplant in previous study [100], the LAGE-1a isoform and NY-ESO-1 could be considered as one “single” CTA for immunotherapy purposes [135].

 Currently, an immunotherapy trial targeting the CTAs MAGE-A3 and NY-ESO-1 in MM patients is in progress (NCT00090493).

## 6. Cell-Based Myeloma Vaccines

 Instead of vaccinating myeloma patients against TAs, an alternative principle aims at stimulating the immune system with the entirety of the myeloma cell's antigens [136]. Such approaches may be implemented by using tumor cell lysates or apoptotic tumor cells as a source for antigens. In a direct comparison, irradiated, apoptotic tumor cells appear to be a superior source for antigen compared to tumor lysates for DC-mediated T cell stimulation [137]. Indeed, stimulation of T cells from the peripheral blood or bone marrow with autologous dendritic cells that had been coincubated with purified, irradiated myeloma cells may give rise to T cell lines with specific IFN-*γ* production and lytic activity of primary autologous tumor cells. In this approach, presentation of antigens from myeloma cell lines by DC s is greatly enhanced by coating of myeloma cells with a specific antibody such as anti-CD138 [138]. Similar results with induction of specific, cytotoxic T cell activity against autologous myeloma cells have also been reported when DCs were loaded with myeloma cells lysed by repetitive freeze-thaw cycles [139].

 Among the leukemia-associated antigens (LAAs), RHAMM, proteinase 3, and WT-1 have been already tested for clinical peptide vaccination [116, 140]. A Phase I/II R3 peptide vaccination for patients with AML, MDS, and MM overexpressing RHAMM was initiated [141]. Patients with a diagnosis of MM were included who fulfilled the following criteria: partial remission (PR) or near-complete remission (NCR) after a high-dose chemotherapy with melphalan and ASCT; immunofixation still positive; free light chains in serum and/or urine were detectable. The patients expressed both RHAMM and HLA-A2 as assessed by RT-PCR and flow cytometry. Authors found a significant increase of specific CD8+ T cells recognizing the RHAMM-R3 peptide in 4/9 patients by ELISpot analysis and/or by tetramer staining. However, due to the number of patients in this Phase I trial no meaningful statistical analysis could be performed.

 The interaction between the CD40 ligand (CD40L) on activated T lymphocytes and CD40 on the APCs has been shown to be crucial for the induction of cytotoxic T lymphocyte (CTL) immunity. CD40−B cells can be generated in large numbers from small amounts of peripheral blood and have the potential to serve as a cellular adjuvant for immunotherapy [142]. The CD40−B cells loaded with tumor lysates induced strong target-specific CTLs, based on large numbers of IFN-*γ*-secreting cells and higher cytotoxic activity against target cells compared to the CD40−B cells without the tumor lysates [142].

 Recently, hTERT (human telomerase reverse transcriptase) was detected in the majority of human malignancies. HTERT can be a target for CT−T lymphocytes in several malignancies including MM *in vitro *[143] and *in vivo* [144]. Kryukov et al. studied antigen-specific and HLA-A2-restricted cytotoxic activity against an ARH77 myeloma cell line *in vitro *[145], when HLA-A2-specific hTERT-derived nonapeptide was used as a TAA. Myeloma-specific cytotoxic activity of hTERT-reactive CTLs was established by repeated stimulation of the CTLs via DCs loaded with hTERT-derived nonapeptide.

 In cancer immunotherapy, including MM, there is no proof that a cancer vaccine has to stimulate a large number of T cells in order to initiate tumor rejection. T cell responses to tumor antigens may be of a low level, and negative results obtained with most *ex vivo* assays may not exclude the beneficial effect of tumor-specific T cells *in vivo*. After stimulation, myeloma-reactive T cells activate and produce IFN-*γ*. Such activated T cells can be efficiently expanded *in vitro *without loss of specificity to the target myeloma antigens. Cytotoxicity of expanded myeloma-reactive T cells was evaluated and demonstrated a strong and myeloma-specific response which, as expected, was mainly CD8+ CTL dependent [146]. Further expansion of sorted myeloma-reactive T cells containing both helper and cytotoxic T cells does not lead to loss of antigen specificity but, rather, leads to potentiation of cytotoxicity, probably via beneficial cytokine production by helper T cells that positively influences further proliferation and the cytotoxic potentiality of CD8+ CTLs. Immunization with MUC1 protein results in activation of CT−T lymphocytes both *in vitro *and *in vivo* [147]. After immunization with this antigen, activated T lymphocytes were separated immunomagnetically and expanded *in vitro *[148]. Specific cytotoxicity of the expanded T lymphocytes was tested against a myeloma cell line [148]. Such an approach can also be useful therapeutically as, after enrichment, myeloma-reactive T cells can be expanded *in vitro *to reach amounts useful and effective in clinical trials. An approach which was recently introduced into the clinical setting relies on adoptive transfer of T cells expressing transgenic T cell receptors (TCRs) with antitumor function; however, there is a critical bottleneck in identifying high-affinity TCR specificities necessary for treatment of various malignancies [149].

 In general, the process of identification and characterization of individual myeloma specific T cell clones can be used as a tool for immune monitoring during cancer treatment.

Depletion of CD25+ regulatory T cells by specific monoclonal antibodies like denileukin diftitox (Ontak; [150]) or addition of toll-like receptor stimulation oligodinucleotides might pave the road for new approaches in the field of peptide vaccination [151]. Moreover, advances are being made in the combination of peptide vaccination with alloSCT [140].

## 7. Vaccines for Myeloma Based on Dendritic Cells

 Preclinical studies have shown that DCs generated from myeloma patients were functional and could efficiently present Id determinants to autologous T cells [83, 152]. DCs pulsed with Id protein can be used to induce the type-1 anti-Id response in myeloma patients. Wen and coworkers [79, 95] reported results from vaccinating an MM patient with autologous Id protein-pulsed DCs generated from blood adherent cells. An immune response against Id was demonstrated in many of the patients. A minor clinical response (25% reduction in the M-component) was observed in one patient and stable disease in the remaining patients. Cull and coworkers [153] reported on vaccinating two patients with advanced refractory MM with Id-pulsed DCs combined with GM-CSF. An anti-Id T cell proliferative response was detected in both patients, which was associated with IFN-*γ* production by the T cells. One patient also had an anti-Id humoral response. Subcutaneous DC vaccination indeed induces better antimyeloma responses than intravenous DC vaccination [154–156].

 DC vaccines can also be produced in the form of fusion of tumor cells with DCs. Vaccination with fusions of tumor cells and DCs is an effective treatment in animal tumor models [157]. In a murine plasmacytoma model, vaccination with DCs fused with mouse 4TOO plasmacytoma cells was associated with induction of antitumor humoral and CTL responses [158]. Immunization with the fusion cells protected mice against tumor challenge and extended the survival of tumor-established mice without eradication of the tumor cells. Addition of IL-12 helped eradicate the established tumor. In a more recent study, human myeloma cells, either primary myeloma cells from patients or a myeloma cell line U266, were fused to human DCs [159]. Fusions with mature rather than immature DCs induced higher levels of T cell proliferation and activation, as assessed by intracellular IFN-*γ* expression, and stronger CTL activity against the tumor cells [160, 161].

 Based on these results, a clinical trial was designed to evaluate the efficacy of vaccinating myeloma patients with a fusion of myeloma cells and autologous mature DCs [159].

 However, patients with MM have DCs that are functionally defective [42]. In order to generate potent functional DCs, alternative methods for blocking some inhibitory signals have been tested [41, 162]. It was reported that the inhibitory factors and abnormal signaling pathways of DCs during maturation with tumor antigen might be responsible for the defective activity of DCs in MM and suggested that the way to overcome these abnormalities is by neutralizing the signaling that would lead to suppressing the immune response [163]. In an attempt to increase DC potency by the use of cytokine combinations, alpha-type-1-polarized DCs (*α*DC1s) that are induced to mature using the *α*DC1-polarizing cytokine cocktail (interleukin (IL)-1*β*, tumor necrosis factor (TNF)-*α*, interferon (IFN)-*α*, IFN-*γ*, and polyinosinic:polycytidylic acid [poly(I:C)]) can be developed to generate strong functional CTLs in several diseases, compared to standard DCs (sDCs) [164]. When mononuclear cells (MNCs) from the BM are used as a source of tumor antigen, the DCs usually show weak antigenicity due to possible contamination with normal cells. To overcome this problem, the previous report demonstrated that DCs pulsed with purified and optimized myeloma antigen could generate potent myeloma-specific CTLs [165]. Recently, the possibility of cellular therapy using autologous *α*DC1 pulsed with the ultraviolet B (UVB)-irradiated allogeneic myeloma cell line, ARH77 with HLA-A0201+, which could generate myeloma-specific CTLs against autologous myeloma cells was investigated [166].

 Vaccination with DC/tumor fusions induces antitumor immunity in the majority of the patients; however, the responses are transient and not always associated with clinical benefit. One potential limiting factor is the regulatory T cells. Developing strategies that promote the expansion of functionally competent tumor reactive T cells and limit the influence of regulatory T cells is necessary to improve the efficacy of the DC/MM fusion vaccine. One approach is vaccination in conjunction with ASCT which facilitates vaccine response by inducing a minimal residual disease state and limiting the inhibitory influence of the myeloma cells. In preclinical models, stem cell transplantation results in the *in vivo* depletion of regulatory T cells, transient loss of tumor mediated tolerance, and enhanced capacity to respond to tumor vaccines [167, 168].

 PD-1 expression is upregulated on T cells isolated from patients with MM, and PD-1 blockade is associated with enhancement of T cell response to the vaccine. Luptakova et al. examined the effect of lenalidomide on T cell activation and polarization, PD-1 signaling, and vaccine-induced responses *in vitro *[169]. *In vitro *exposure to lenalidomide results in enhanced T cell activation in response to direct ligation of the costimulatory complex and stimulation by the DC/myeloma fusion vaccine, suppressed T cell expression of PD-1 and regulatory T cells, 2 critical pathways responsible for tumor-mediated immune suppression. This is the first demonstration of an interaction between lenalidomide and the PD-1/PDL-1 pathway. These findings support the development of cellular immunotherapy in conjunction with lenalidomide, including its use with the DC/myeloma fusion vaccine [169]. Lenalidomide resulted in greater degree of Th1 polarization as manifested by a 2-fold increase in the percentage of CD8+ T cells expressing IFN-*γ* (*P* = 0.02) and a decrease in the percentage of regulatory T cells from 6.88% to 3.13% (*P* = 0.02). In addition, the percentage of NK cells expressing IFN-*γ* was 5-fold greater (*P* = 0.03) in the presence of lenalidomide.

Lastly, Luptakova et al. found that vaccination with fusion-mediated stimulation of autologous T cells in the presence of lenalidomide resulted in an increase in the percentage CD4+ and CD8+ T cells expressing IFN-*γ* (5.35% to 8.79%, *P* = 0.06, and 6.37% to 9.85%, *P* = 0.03, resp.). The proportion of regulatory T cells decreased from 9.57% to 4.43% in the presence of lenalidomide (*P* < 0.01). As with nonspecific stimulation, PD-1 expression on CD4+ cells in the presence of lenalidomide decreased from 24% to 19%. In concert with these findings, exposure to lenalidomide resulted in increased cytotoxic T lymphocyte-mediated lysis of autologous tumor targets (from 25% to 36%). At this time several clinical studies recruit patients for clinical investigation [169].

## 8. NK Cells

 DCs and NK cells reciprocally activate each other during the immune response. DCs can stimulate NK cells to produce interferon-*γ* (IFN-*γ*) and to expand NK cells *in vitro *[170, 171]. Reversely, the DCs can be activated by NK cells to increase the production of cytokine, costimulatory molecules expression, and T cell stimulation, resulting in a more efficient Th1-type polarization and CTL generation [172, 173]. Nguyen-Pham et al. investigated the possibility of generating potent DCs through stimulation with NK cells in the presence of different cytokines in order to induce myeloma-specific CTLs against MM *in vitro *[174]. They demonstrated that potent DCs can be generated by stimulation with NK cells, as activator helper cells, in the presence of TLR3 agonists, IFN-*γ*, and IL-2. NK cell-stimulated DCs exhibited high expression of costimulatory molecules and high production of IL-12p70 [174]. These DCs induce high potency of Th1 polarization and exhibit a high ability to generate myeloma-specific CTL responses. These results suggest that functionally potent DCs can be generated by stimulation with NK cells and may provide an effective source of DC-based immunotherapy in multiple myeloma [174].

 Recently, it has been shown that autologous NK cells from myeloma patients expanded *ex vivo* with IL-2 displayed significant cytotoxic activity against primary autologous plasma cells [38]. However, *ex vivo* or *in vivo* expansion of the NK cells with IL-2 carries a dose-limiting toxicity.

The potential of tumor-activated (TaNK) cells to induce lysis has been explored [175]. Recent study was designed to assess the relative function *in vitro *of NK and TaNK cells from MM patients compared to normal healthy controls in the lysis of tumor cell lines and freshly isolated primary autologous and allogeneic MM cells [175]. In this study, the authors demonstrated that TaNKs from myeloma patients can induce a substantial lysis of myeloma cell lines as well as autologous and allogeneic freshly isolated BM malignant plasma cells. This was in accordance with the degree of killing reported in the study by Alici et al. [38], where NK cells underwent *ex vivo* expansion with the addition of IL-2. This potential is not affected either by the disease status or by the antimyeloma treatment, including novel agents and dexamethasone. These findings provide information for the use of TaNK cells in MM therapy and particularly in combination with the novel agents.

 Modulation of inhibitory and activating NK receptor ligands on tumor cells represents a promising therapeutic approach against cancer, including MM. Proteasome inhibitors, in particular lactacystin, that most probably target inhibitory KIR ligand class I on the MM tumor cells may contribute to the activation of cytolytic effector NK cells in vitro, enhancing their antimyeloma activity [176].

 Several reports showed a reciprocal relationship between NK and Tregs [177]. In addition Tregs could suppress the function of NK cells [178]. A unique mouse model of MM, namely, 5T2MM-bearing mouse, was useful for elucidating the pathophysiological mechanisms underlying the disease [179]. Depletion of Tregs, a proposed strategy in cancer immunotherapy, was tested using cyclophosphamide (CY). Low-dose CY, which selectively depletes Tregs, decreased MM incidence, in contrast to high-dose CY, which was generally cytotoxic, and did not reduce MM incidence. On the other hand, the number and function of NK cells could be recovered, the production of IFN-*γ* was enhanced, and DCs could continue their differentiation and become mature and ready for activation [179].

Th1-type cytokines invariant natural killer T (iNKT) cells have been shown important in initiating antitumor immune responses. Through the production of IFN-*γ*, iNKT cells can stimulate the activation of downstream effectors including T cells, NK cells, dendritic cells, and macrophages and increase NK and T cell proliferation and cytotoxicity through IL-2 production [180–183]. However, both quantitative and qualitative defects of iNKT cells in advanced MM hamper their antitumor effects. Song et al. developed a novel immunotherapeutic strategy directed at the activation and expansion of Th1-polarized iNKT cells from MM patients [184]. Functional iNKT cell lines were generated from MM patients with a-GalCer-pulsed DCs and further improved by lenalidomide. These results provide the preclinical feasibility and rationale for iNKT cell-mediated immunotherapy in MM [184].

## 9. Monoclonal Antibodies in the Treatment of MM

### 9.1. General Considerations

 A wide range of antigens may ultimately be targeted in MM therapy, including those involved in cell survival, antiapoptotic pathways, cell-to-cell communication, angiogenesis, and interactions between MM cells and bone marrow stromal cells (BMSCs) and/or other cells in the BM microenvironment [26, 185]. These potential targets include signalling molecules, cell surface receptors and other cell surface proteins, plasma cell growth factors, and mediators for adhesion. Ideally, a useful target for mAb-based MM therapy should be expressed exclusively on the majority of the MM cells (or other target cells such as those involved in angiogenesis) [26]. The clinical efficiency of most therapeutic antibodies is based on their capacity to recruit and activate cytotoxic effector mechanisms of the innate immune system. This occurs either by engagement of activating Fc receptors expressed on NK cells or macrophages on the tumor cell surface leading to ADCC or by activating the complement cascade through tumor cell-bound antibodies (CDCC). Other possible mechanisms include interference with ligand binding (e.g., growth factor or G-protein coupled receptors) and the use of mAbs as targeted “carriers” of cytotoxic agents [4].

**CD20**

Clinical studies of rituximab therapy for MM have been disappointing, as only a few of the patients showed minimal response [186]. The failure of rituximab in this setting is potentially attributable to the small number of MM patients (estimated at 13–22%) who express CD20 in primarily a low proportion of plasma cells. Another mechanism that may render MM refractory to rituximab is the possibility that MM cells express increased levels of complement-inhibiting proteins, thus reducing the effectiveness of CDC. In addition, Fc-c receptor polymorphism may limit the effectiveness of ADCC as a killing mechanism. Finally, the use of rituximab for MM may induce a selective loss of the CD20 expression [186]. Although it is conceivable that rituximab may be useful for some carefully-selected MM patients, such as t (11; 14) translocation patients, who exhibit relatively high CD20 expression [187], it is unlikely to be of value for the majority of cases.

**CD40**

CD40 is a transmembrane protein belonging to the TNF-*α* superfamily, normally expressed in the resting cell types, with the highest levels of expression found in B and DCs [188–190]. CD40 is expressed at high levels on the surface of MM cell lines and primary MM cells [191]. The binding of CD40 to its natural ligand determines its functional activation that, in turn, induces diverse biologic events including MM-cell proliferation and migration via the PI3K/AKT/-NF *κ*B signaling pathway. CD40 is also expressed by BMSCs, and upon activation it triggers the secretion of IL-6 and VEGF [192–194]. Thus, inhibition of CD40−CD40L interaction could exert antimyeloma activity through the blockage of several critical pathways in MM or in BMSCs. Monoclonal antibodies developed against CD40 (SGN-40, CHIR-12.12) [195] have shown a modest cytotoxic activity against MM cell lines and primary MM cells when used as single agents for treatment [196]. The mechanisms of action rely on the inhibition of CD40−CD40L interaction and activation as well as on ADCC [197, 198]. Although earlier trials in NHL and MM were promising, a Phase II NHL trial comparing therapy with the anti-CD40 antibody SGN-40 with existing treatments alone was stopped because of lack of efficacy. Horton et al. described the characterization of XmAbCD40, a humanized anti-CD40 antibody with increased Fc*γ*R binding that facilitates highly enhanced ADCC against B-lymphoma, leukemia, and MM cell lines and against primary tumor cells from patients with CLL and plasma cell leukemia (PCL) [199]. XmAbCD40 shows nearly 2 orders of magnitude increased binding to Fc*γ*RIIIa and 1 order of magnitude increased binding to Fc*γ*RIIa. The increased affinity for Fc*γ*RIIIa translated into dramatically increased NK cell-mediated ADCC. Results were consistent in several cell lines expressing different levels of CD40 antigen as well as in patient-derived primary tumors [199].

The observation that SGN-40-induced MM cell death is enhanced by lenalidomide [200] led to its evaluation in a Phase I study in combination with lenalidomide and dexamethasone in patients with relapsed or refractory MM; an overall response (OR) of 39% (13/36) was seen, with some activity noted in patients receiving prior lenalidomide [201]. Phase I clinical trials of SGN-40 in combination with other agents are currently ongoing [202].

Lucatumumab is a fully human anti-CD40 MAb that inhibits MM cell growth *in vitro*, even when MM cells are cocultured with BMSCs. Animal studies have shown that the primary cytotoxic mechanism is ADCC [198]. However, a Phase I study of lucatumumab in patients with relapsed/refractory MM was terminated because of minimal biological and clinical activity (NCT00231166).

***CS1 *(CD2 Subset 1, CRACC, SLAMF7, CD319, or 19A24)**

CS1 is a cell surface glycoprotein of the immunoglobulin-gene superfamily with high expression on the surface of MM cell lines and on plasma cells from MM patients [203]. It is not expressed on other normal tissues [203]. The role of CS1 is not well understood; however, there is evidence that it participates in promoting and supporting MM cell adhesion, tumor growth, and proliferation through interactions with bone marrow stromal cells mediated by c-maf pathway activation [203, 204].

A humanized mAb developed against CS1 elotuzumab (HuLuc63) has been proven to induce significant antimyeloma activity both *in vitro *and *in vivo* [203, 205]. *In vitro*, the employment of bortezomib has been shown to increase HuLuc63-induced ADCC [206]. *In vivo* injection of the mAb significantly induced tumor regression in xenograft myeloma mouse models [203]. Based on these results, Phase I clinical trials are underway to evaluate the safety and toxicity of the HuLuc63 in myeloma patients [207]. Elotuzumab demonstrated acceptable toxicity but its antitumor activity was only modest: no responses were seen, although elotuzumab did induce stable disease (SD) in six of 19 patients [208]. Clinical studies of elotuzumab combined with either lenalidomide plus dexamethasone or with bortezomib were therefore initiated and are showing considerable promise. In a preliminary analysis of an ongoing phase I study of elotuzumab plus bortezomib, the ORR (partial pesponse (PR) or better) was 48% for 27 evaluable patients, and responses were achieved for several bortezomib-refractory patients. A clinical response was seen in 17/27 (63%) patients. The response rate was lower among heavily pretreated patients (>3 prior therapies) and the median time to progression was 9–46 months [209]. In a preliminary analysis of an ongoing Phase Ib combination study with lenalidomide and dexamethasone, the ORR was 82% for all treated patients (*n* = 28), 96% for lenalidomide-naïve patients (*n* = 22), and 82% among patients who had been refractory to their most recent treatment (*n* = 11) [210]. In a Phase II study of the same combination, the ORR was 85% for evaluable patients (22/26), and the remaining four patients had SD; 31% achieved either a complete remission (CR) or very good partial response (VGPR) [211]. Elotuzumab is therefore the first mAb in combination with either bortezomib or lenalidomide and dexamethasone to demonstrate clinically meaningful efficacy in relapsed/refractory MM.

***CD138 (*syndecan-1)**

syndecan-1 is a member of the syndecan family, which includes cell-surface heparan sulfate proteoglycans involved in cell adhesion, maturation, and proliferation [212]. The high levels of heparan sulfate in the tumor microenvironment resulting from syndecan-1 shedding also act as positive regulators that condition the microenvironment for robust tumor growth. This antigen is usually absent on haematopoietic cells; conversely it is frequently expressed on normal and myeloma plasma cells. When present at high levels in the serum, syndecan-1 is an independent indicator of poor prognosis [213–215]. Studies in animal models have shown that high levels of soluble syndecan-1 enhance both the growth and metastasis of tumors [216]. Syndecan-1 has been explored as a candidate antigen for antibody targeting of toxins to the tumor cell surface [138, 217–219].

**CD74**

CD74 expression has been demonstrated for more than 90% of B-cell malignancies [220] and for a high percentage of MM cases (around 80%). To assess CD74 as a therapeutic target, an anti-CD74 mAb, LL1, has been developed [221]. LL1 activity hardly relied on ADCC and CDC mechanisms. This feature makes it feasible to use drug- and toxin-conjugated or radiolabelled forms of LL1 instead of unconjugated ones. hLL1-dox (IMMU-110), for example, is an immunoconjugate composed of doxorubicin conjugated to hLL1 IMMU-110 which has been evaluated in preclinical studies with non-Hodgkin's lymphomas and MM models, resulting in the achievement of an excellent therapeutic response [221, 222]. IMMU-110 appeared to be safe in a monkey model of MM [222]. IMMU-110 is being evaluated in a Phase 1/2 study (NCT00421525), and a Phase 2 study is currently ongoing (NCT01101594).

**CD162**

CD162 has been found to be constitutively expressed in indolent and aggressive plasma cell disorders, including MM, and in normal plasma cells [223]. The anti-CD162 blocking mAb KPL1 has been recently tested *in vitro.* KPL1-mediated CD162 crosslinking was proven to induce death MM cells, in MM cell lines and in neoplastic cells purified from patients, mainly by activating the mitochondrial pathway of apoptosis [224]. KPL1 also mediated a significant induction of ADCC and to a lesser extent complement-dependent cell lysis. Its action could be strongly enhanced by adding blocking mAbs against the complement regulatory proteins CD46, CD55, and CD59 highly expressed on the surface of MM cells [224].

**CD66**

CD66 proteins are expressed in a number of isoforms, which have a wide range of biologically important functions including cell adhesion, cellular migration, pathogen binding, and activation of signalling pathways. This was utilized in recent Phase I and II clinical trials [225, 226] for targeted delivery of radiotherapy to the BM as a part of the conditioning regimen for transplantation in acute leukemias and MM. The expression of CD66a but no other CD66 isoforms on two human myeloma cell lines (U266 and ARH77) and on plasma cells from patients with MM [227] may help in the optimization of future radioimmunotherapeutic strategies by supporting the use of a monoclonal CD66a antibody for targeted radiotherapy in patients with MM [227].

***Beta2-Microglobulin *(*β*2M)**

*β*2M is a nonglycosylated polypeptide, which is a part of the MHC class I molecule on the surface of nucleated cells [228]. *β*2M is normally found in body fluids, but elevated serum levels are present in hematological malignancies [229], including MM [230], and correlate with a poor prognosis. The mAbs against *β*2M have a remarkably strong apoptotic effect on myeloma cells [231]. The anti-MM activity of this antibody was confirmed by *in vivo* in a MM xenograft mouse model experiment which demonstrated selective effect on tumor cells without damaging BM hematopoietic cells of implanted human bone or murine organs expressing *β*2M/HLA-A2 molecules [231, 232]. Therefore, such mAbs offer the potential for a therapeutic approach to hematological malignancies [233].

**CD38**

Under normal conditions, CD38 is expressed at relatively low levels on lymphoid and myeloid cells and in some tissues of non-hematopoietic origin [234]. In the past, several Abs to human CD38 have been generated. These Abs induce killing of neoplastic B cell lines [235, 236]. The relatively high expression of CD38 on all malignant cells in MM [237, 238] in combination with its role in cell signaling suggests CD38 as a potential therapeutic Ab target for the treatment of MM. Two CD38 mAbs are currently in clinical development: a humanized mAb (SAR650984) and a human mAb (daratumumab) [239]. Daratumumab was found to effectively kill MM tumor cells by ADCC and CDC. It was active at low concentrations in a SCID mouse xenograft tumor model. Daratumumab is currently in a Phase I/II safety and dose finding study for the treatment of MM (NCT00574288). Results of this preliminary study are awaited with interest, with early reports suggesting favourable tolerability and disease stabilization for some patients [28]. In a series of experiments using a CD38+ MM cell line, purified MM cells, and full BM mononuclear cells (BM-MNC) of MM patients containing 2–50% malignant plasma cells, van der Veer et al. demonstrated that lenalidomide significantly improves daratumumab-dependent lysis of MM cells [240].

**PD1**

Accumulating experimental evidence indicates that PD1 is a coinhibitor and primarily involved in the regulation of T cell and NK-cell responses. Anticancer immunotherapy based on antibodies directed against the B7 family of receptors, particularly the B7 homologue 1 (B7-H1)-programmed death 1 (PD1) system, suggests a promising novel approach for promoting immune responses against cancer as well as breaking up tumor resistance and dormancy. CT-011 is a humanized IgG1 mAb that modulates the immune response. Interaction of CT-011 with PD-1 leads to stimulation of the NK-cell activity and to extended survival of effector/memory T cells, culminating in the enhancement of antitumor immune response and the generation of tumor-specific memory cells [241]. CT-011 was recently administered to patients with various hematologic malignancies, including MM at an advanced stage of their disease and following chemotherapy and/or stem cell transplantation [242]. Clinical benefit was observed for 33% of the patients with one CR [242]. Currently a new clinical study recruits patients to evaluate the efficacy and safety of CT-011 following autologous transplantation and a Phase II study to determine if cellular immunity is induced by treatment with CT-011 and DC/myeloma fusion cells in conjunction with stem cell transplantation (NCT01067287).

**IL-6**

IL-6 has been recognized as a key cytokine in the development and progression of MM, exerting antiapoptotic activity and multiple additional effects within the BM. IL-6 is produced predominantly by BMSCs and is upregulated by multiple cytokines [185]. Both IL-6 and its receptor, IL-6R, are potential targets for mAb-based intervention. A chimeric anti-IL-6 mAb, siltuximab (CNTO 328), enhances dexamethasone-induced cytotoxicity in MM cell lines, and in MM cells from patients refractory to dexamethasone therapy, it also enhances the cytotoxicity of the bortezomib plus dexamethasone combination [243]. Siltuximab is currently being evaluated in MM in multiple single-arm and randomized Phase II studies, either alone or in combination with bortezomib (NCT01219010, NCT00402181, NCT00911859, NCT00412321, NCT00401843). Preliminary results in combination with bortezomib have shown promise, with a 57% objective response rate (ORR), although grade 3+ haematological toxicities were somewhat common [244]. A Phase III study of siltuximab or placebo in combination with bortezomib and dexamethasone is underway (NCT01266811).

A murine anti-IL-6 mAb, BE-8, has been evaluated in combination with dexamethasone and high-dose melphalan as a conditioning regimen for ASCT. The combination induced a response in 13 of 16 patients (81%) and a CR in 6 patients (37.5%). The overall response (OR) was similar to historical controls by the same group of high-dose melphalan; however, the CR rate appeared to be higher and was correlated with IL-6 neutralization [245]. In a subsequent prospective, multicentre randomized trial by the same group, the addition of BE-8 to the melphalan plus dexamethasone conditioning regimen showed no improvement in response or survival rates for patients with high-risk MM [246].

Tocilizumab is a humanized anti-IL-6 mAb currently approved for rheumatoid arthritis in several countries, and for the Castleman disease in Japan, has demonstrated efficacy in a murine MM model [247] and is currently being evaluated clinically in MM. Another anti-IL-6 mAb, 1339, has demonstrated activity on MM cell lines (cocultured with BMSCs) *in vitro* and in murine xenograft MM models; it is not yet being evaluated clinically [248].

**VEGF**

VEGF is a key cytokine that promotes angiogenesis in a variety of tumour types. Bevacizumab, a humanized anti-VEGF mAb, is currently indicated for treatment of colorectal cancer. In a Phase II study in patients with relapsed/refractory MM, seven out of 10 patients responded partial response (PR) to bevacizumab in combination with low-dose dexamethasone and lenalidomide [249]. An additional phase II study of the same combination reported similar results in a larger patient population (OR 19/27, 70%) [250], noting that this response rate was not significantly different from that seen in the pivotal Phase III trial of lenalidomide plus dexamethasone (61%) [251]. An additional Phase II study of this combination is currently recruiting patients (NCT00410605), and the drug is also being evaluated in combination with bortezomib (NCT00464178, NCT00473590).

**GM-2**

GM-2 is a ganglioside expressed on MM cells. A humanized anti-GM-2 mAb, BIW-8962, has demonstrated *in vitro *killing of MM cell lines and *in vivo* effectiveness in mouse xenograft models, with ADCC and CDC the most prominent cytotoxic mechanisms [252]. BIW-8962 is being evaluated as monotherapy in a Phase I/II study for patients with relapsed/refractory MM (NCT00775502).

**CD200**

CD200 is a highly conserved transmembrane glycoprotein expressed on a wide range of cell types; however, expression of the receptor for CD200 (CD200R1) is apparently limited to APC of myeloid lineage and certain T cell populations and is thought to deliver inhibitory signals. The expression of the CD200 gene by MM cells has been found to be a predictor of poor prognosis in patients with MM [253]. ALXN6000 is a humanized anti-CD200 mAb that is currently being evaluated in a Phase 1/2 study in patients with MM or B-cell CLL (NCT00648739), with results expected in the near future.

**Killer Cell Immunoglobulin-Like Receptors (KIRs)**

KIRs are receptors expressed on natural killer (NK) cells and a subset of T cells and function as key regulators of NK cell activity [254]. IPH 2101 (anti-KIR) is a fully human monoclonal antibody blocking interaction between KIR inhibitory receptors on NK cells with their ligands. By blocking these receptors, it facilitates activation of NK cells and, potentially, destruction of tumor cells by the latter. Several studies are currently underway in smoldering and first-relapse MM (NCT01222286, NCT01217203, NCT00999830, NCT01248455) and safety and tolerability results are expected later in 2011 for a Phase 1 study in relapsed or refractory MM (NCT00552396).

## 10. Monoclonal Antibodies and Highly Cytotoxic Compounds

 The immune system of MM patients is impaired by the disease or by cancer treatments. Along with efforts to develop functional antibodies, substantial efforts are underway to develop therapies using antibodies conjugated with potent cytotoxic agents. A variety of highly cytotoxic compounds are being evaluated for antibody-based delivery, including calicheamicin, doxorubicin, taxanes, maytansinoids, dolastatins, and CC-1065 analogs [255–258]. Immunoconjugate IMGN901 (BB-10901; huN901-DM1) is composed of a humanized monoclonal antibody that binds with high affinity to CD56 conjugated with the cytotoxic maytansinoid DM1 through a disulfide linkage [258]. Upon binding to a target tumor cell, the antibody-maytansinoid conjugate is internalized by natural processes, where the conjugate is metabolized and active maytansinoid metabolites are released [259]. Within the hematopoietic compartment, while CD56 expression is normally restricted to NK cells and a subset of T lymphocytes [260, 261] and is absent from normal plasma cells [262], it is strongly expressed on MM cells in the majority of MM patients [263–266]. Tassone et al. demonstrated the activity of IMGN901 against CD56+ MM cells both *in vitro *and *in vivo*. Target-dependent cytotoxicity was shown in cocultures of CD56+ and CD56− cells [263]. Treatment with IMGN901 in a human MM tumor xenograft model in immunocompromised mice showed that the immunoconjugate was effective in both a minimal and bulky disease setting. The clinical evaluation of IMGN901 was initiated with a Phase I study in patients with relapsed or relapsed/refractory MM who failed at least one prior therapy and have CD56+ MM (NCT00346255) [258]. Additive to synergistic activity has been observed in combinations of IMGN901 with lenalidomide, bortezomib, or melphalan in MM xenograft models [267, 268].

 BT062 is a chimeric mAb conjugated to maytansinoid derivatives that demonstrates *in vitro *cytotoxicity and inhibition of MM cells in mouse xenograft models, apparently via apoptotic mechanisms; BT062 also inhibits the adherence of MM cells to BMSCs and abrogates the protective effects exerted by growth factors and BMSCs on MM cells [269]. The local release of potent maytansinoid moieties from target cells and uptake into nearby nontarget cells is the proposed mechanism for this activity [269] and may have an important impact on BT062 efficacy through eradication of tumor cells that heterogeneously express CD138 or disruption of the tumor microenvironment by elimination of tumor stromal cells. A Phase I dose finding study of BT062 for patients with relapsed/refractory MM is underway (NCT00723359), and an additional Phase I/IIa study is ongoing but not recruiting patients with advanced MM (NCT01001442).

## 11. Immunotherapy Approaches Targeting Microenvironment and the Neoplastic Niche of MM

The BM microenvironment encompasses a wide spectrum of cell types and extracellular matrix proteins, including fibronectin, collagen, laminin, and osteopontin. Multistep genetic and microenvironmental changes lead to the transformation of plasma cells into a malignant neoplasm. Genetic abnormalities alter the expression of adhesion molecules on myeloma cells, as well as responses to growth stimuli in the microenvironment [270].

 A cardinal clinical feature of MM is the presence of osteolytic bone lesions. Myeloma cells disrupt the delicate balance between bone formation and bone resorption [271, 272]. The inhibition of the Wnt pathway suppresses osteoblasts, whereas the amplification of the RANK pathway and the action of macrophage inflammatory protein 1 *α* (MIP1*α*) activate osteoclasts [271]. The induction of proangiogenic molecules (e.g., VEGF) enhances the microvascular density of bone marrow and accounts for the abnormal structure of myeloma tumor vessels [273]. Various clinical observations [274] and experimental studies [275, 276] have linked the level of the MM bone disease with the disease burden. Tumor cells and stromal cells interact via adhesion molecules and cytokine networks to simultaneously promote tumor cell survival, drug resistance, angiogenesis, and disordered bone metabolism. In addition, the amounts of several of immunologically active compounds increase including TGF-b, IL-10, IL-6, VEGF, Fas ligand, MUC-1, cyclooxygenase (COX)-2, and related prostanoids and metalloproteinases [277].

 In addition to therapy directed at MM cells and tumour promoting interactions, some efforts have been devoted to mAb therapy directed against the development of end-organ complications; to date, these efforts have been restricted to the suppression of myeloma-related bone disease.

 Angiogenesis is considered a hallmark of MM progression. As indicated before in patients affected by MM syndecan-1, a heparan sulphate proteoglycan is overexpressed by myeloma cells in the BM and peripheral blood [212]. The high levels of heparan sulfate in the tumor microenvironment resulting from syndecan-1 shedding also act as positive regulators that condition the microenvironment for robust tumor growth. For example, heparan sulfate binds to and promotes the activity of important angiogenic growth factors such as fibroblast growth factor-2 (FGF-2) and VEGF [278, 279]. Recent research has shown that syndecan-1 could also be involved in the modulation of the growth and survival of endothelial cells (ECs) within the BM microenvironment [280]. Enzymatic remodeling of heparan sulfate proteoglycan structure and function within the tumor microenvironment is emerging as an important mechanism for dynamic regulation of tumor growth [281]. There are three forms of enzymatic remodeling of heparan sulfate proteoglycans that are known to occur in myeloma, and other tumors, sulfatases, sheddases, and heparanase, which are active within the tumor microenvironment, point out the importance of regulated remodeling of heparan sulfate proteoglycans [216, 281–284]. Certain heparinase gene SNPs may contribute to basal heparanase gene expression. Alterations in this gene are an important determinant in the pathogenesis of ALL, AML, and MM [285]. Dynamic regulation of heparan sulfate structure by sulfate 6-*O*-endosulfatases (Sulfs) present within the tumor microenvironment can have a dramatic impact on the growth and progression of malignant cells *in vivo *[283].

 The high serum level of shed syndecan-1 has been associated with an unfavourable prognosis [213, 215].

Hence, the designing of novel agents that regulate the remodeling processes of heparan sulfate proteoglycans or inhibiting of VEGF as discussed previously represents a new opportunity for therapeutic control of malignant cell growth. Huang and zhan investigated the effect of VEGF antisense (AS) RNA on proliferation and apoptosis in myeloma cell line U266 as well as on angiogenesis in endothelial cell ECV304 and to explore the feasibility of gene therapy for MM using VEGF antisense RNA [286]. VEGF121 cDNA was inserted into a multiple clone site of eukaryotic expression vector pIRES2-EGFP to construct the recombinant plasmid AS-VEGF. The recombinant plasmid was transfected into a human myeloma cell line U266. Expression of VEGF mRNA and protein decreased more significantly in U266 cells transfected by AS-VEGF than that in control group. VEGF antisense RNA can inhibit the expression of VEGF gene in U266 cells, thereby inhibits the proliferation of U266 cells, increases the apoptosis of U266 cells, and inhibits angiogenesis *in vitro *[286].

 Another novel therapeutic concept related to the microenvironment is the introduction of antiadhesion strategies. Podar et al. evaluated the therapeutic potential of the new-in-class molecule-selective adhesion molecule (SAM) inhibitor Natalizumab, a recombinant humanized IgG4 monoclonal antibody, which binds integrin-*α*4, in MM [287]. Natalizumab, but not a control antibody, inhibited adhesion of MM cells to non cellular and cellular components of the microenvironment as well as disrupted the binding of already adherent MM cells. Moreover, natalizumab also blocked VEGF- and insulin-like growth factor 1 (IGF-1)-induced signalling sequelae triggering MM cell migration. Natalizumab not only blocked tumour cell adhesion but also chemosensitized MM cells to bortezomib, in an *in vitro *therapeutically representative human MM-stroma cell coculture system model.

 Some MM cells that harbor oncogenic translocations remain dependent on the stroma for their survival, while others acquire additional mutations which affect NF-*κ*B pathways and remove their reliance on the bone marrow microenvironment [288]. Mutations affecting the activation of NF-*κ*B-inducing kinase (NIK) have been identified in MM samples and cell lines, suggesting that NIK could be an important target for therapy of MM. The majority of MM samples display high constitutive NF-*κ*B activity and up to 20% results from mutations in NF-*κ*B signaling components, including NIK. Inhibition of NIK may be an effective therapeutic for some MM cases. There are several new agents under investigation that induce apoptosis of myeloma cells. Celastrol is a quinone methide triterpene derived from the medicinal plant *Tripterygium wilfordii*, acts by NF-*κ*B pathway, and induces cell cycle arrest at the G1 phase followed by apoptosis in human myeloma cell line U266 cells [289]. Several studies have showed that miRNAs play important roles in the regulation of cell proliferation, differentiation, and apoptosis [290, 291]. The deregulation of miRNAs expression contributes to tumorogenesis by modulating oncogenic and tumor suppressor signaling pathways.

## 12. Receptor Activator of Nuclear Factor Kappa-B Ligand (RANKL)

 RANKL promotes bone loss in osteoporosis and contributes to the development of bone lesions in MM. The inhibition of RANKL may directly impact myeloma cells that express RANK and have a therapeutic role in the treatment of MM. The fully human anti-RANKL mAb, denosumab, has demonstrated some efficacy in a Phase II study of patients with plateau-phase or relapsed MM, including suppression of the bone turnover marker serum C-terminal telopeptide of type 1 collagen (sCTx) [292]. Denosumab is currently being compared with zoledronic acid (the standard of care for prevention of bone disease in several cancers) in patients with advanced cancers or MM in a randomized Phase III trial (NCT00330759); results in the MM cohort have thus far been mixed although positive in other cancers; future trials are planned in MM to better define its role.

## 13. Dickkopf-Related Protein 1 (DKK1)

 The canonical Wnt pathway plays an important role in controlling proliferation, differentiation, and survival of OBs. In MM, high serum DKK1 levels were correlated with focal bone lesions [293]. The DKK1 produced by MM cells can inhibit the differentiation of OB precursor cells [293] and bone formation *in vitro *[294] through a DKK1-mediated attenuation of Wnt3a-induced stabilization of *β*-catenin [295]. These findings confirm DKK1 as an important regulator of bone formation in the bone microenvironment. The broad expression in myeloma but highly restricted expression in normal tissues, together with its functional roles as an OB formation inhibitor and a potential myeloma growth enhancer, make DKK1 an ideal and universal target for immunotherapy. DKK1 (peptide)-specific CTLs can effectively lyse primary myeloma cells *in vitro *[296]. A fully human anti-DKK1 mAb, BHQ880, has demonstrated improvement in the bone parameters in murine models and also appears to have direct effects on the MM cell growth, possibly via interactions with the BMSCs and the IL-6-related pathways [297, 298]. BHQ880 is being evaluated in combination with zoledronic acid in a Phase 2 study in patients with relapsed/refractory MM (NCT00741377), and studies in early MM (i.e., smoldering MM) are also underway.

## 14. Biphosphonates-Activated T Cell-Based Immunotherapy

 Aminobiphosphonates, such as pamidronate and zoledronate, were originally developed for osteoporosis but are increasingly used for cancer therapy. They have been shown to activate V*γ*9V*δ*2 T cells, and the activated cells were functionally characterized *in vitro *and *in vivo* [299–302]. *In vivo* study, evaluated administration of low-dose IL-2 in combination with pamidronate to patients with low-grade non-Hodgkin lymphoma or MM, showed that only patients with significant *in vivo* proliferation of *γδ* T cells responded to treatment [303]. Abe et al. [304] in a clinical phase I study evaluated the clinical and immunological effects of zoledronate-activated V*γ*9V*δ*2 T lymphocyte-activated killer (LAK). Six patients with MM received no antimyeloma therapy in the preceding 2 months and during the study period received four biweekly intravenous infusions of zoledronate-activated V*γ*9V*δ*2 T LAK cells generated from the culture of PBMCs in the presence of zoledronate and IL-2. This showed that administration of zoledronate-activated V*γ*9V*δ*2 T LAK cells, a safe and immunotherapy for MM patients, is promising, and zoledronate-activated V*γ*9V*δ*2T cells warrant further clinical investigations.

## 15. Changes in Mesenchymal Stromal Cells from Multiple Myeloma Patients

 BM-derived mesenchymal stromal cells (MSCs) are precursors of OBs and differentiate preferentially into bone-forming cells both *in vitro *and *in vivo*. MM cells were suggested to target MSCs thereby diverting their functions to serve the MM cells. This idea led to studies of the functions of MSCs derived from MM patients (MM-derived MSCs) compared to those of healthy individuals; it was suggested that MSCs from myeloma patients exhibit defective functions [68–70]: MM-derived MSCs were reported to exhibit decreased colony-forming unit number [70], growth impairment [70], reduced osteogenic differentiation [68], and increased IL-6 secretion [68, 70]. However, these observations were not reproducible in all reports [68–70]. Some authors focused on toll-like receptor (TLR) ligands and on the cytokine epidermal growth factor (EGF). They [305–310] have shown that TLR activation modulates MSC proliferation, migration, and differentiation. However, MM-derived MSCs exhibited reduced activation of extracellular signal-regulated kinases (ERK1/2) and may therefore represent a general property of this signaling pathway in MM-derived MSCs. These altered responses persisted in MSCs from MM patients following extended culture and passaging *in vitro*, indicating that these cells are permanently modified. Activation of MAPK pathway contributes to drug resistance, growth, and survival [311]. MSCs derived from MM patients have been shown to exhibit different gene expression profiles when compared to control MSCs [68, 312]. Furthermore, these MSCs have been suggested to be genomically altered [313]. MM-derived MSCs are intrinsically and permanently modified. The treatment of the disease may therefore require not only the elimination of the tumor cells but concomitantly treatment or replacement of stromal elements.

## 16. Immunotherapy after Autologous Stem Cell Transplantation for MM

 A major area of investigation is to develop strategies to elicit myeloma-specific immune responses that will selectively eliminate malignant cells and eradicate residual disease following ASCT. High-dose melphalan induces severe and persistent immunosuppression characterized by a delayed recovery of CD4 T cells that remain below normal counts for months to years after ASCT [314, 315], a restricted T cell repertoire [316], and impaired T cell functions including an increased susceptibility to apoptosis [317], a reduced proliferation intensity upon stimulation with mitogens or defined antigens and a default in Th1 cytokine production that lasts at least one year after ASCT in patients with MM [318, 319]. The B-cell immune response is also altered after ASCT since levels of plasma antibodies after one recall vaccination are below those found in healthy donors [315]. T cell functions are impaired after transplantation in patients with MM despite a recovery of normal numbers of T lymphocytes [317–319]. In theory, the posttransplantation phase should be highly amenable to the application of immunotherapy because of a lower tumor burden. However, after high-dose therapy, the immune system is characterized by immune cell depletion and impaired function that may last for years [314]. The therapeutic induction of rapid lymphocyte recovery consistents that unmanipulated lymphocyte levels in patients with myeloma correlate to event-free survival (EFS) [320–323]. Rapoport et al. have developed a strategy for inducing an effective antitumor immune response during the posttransplantation period and to control or eliminate residual disease [324]. The authors hypothesized that enhanced numeric and functional recovery of T cells might provide a platform for posttransplantation tumor vaccine immunotherapy. The autologous T cells were costimulated with paramagnetic beads that deliver CD3 and CD28 signals designed to reverse T cell anergy [325–328]. Patients with myeloma received costimulated autologous T cells after autotransplantation, along with immunizations with a 7-valent pneumococcal conjugate vaccine (PCV; Prevnar; Wyeth) [324, 329]. In addition, patients who were positive for human leukocyte antigen A2 (HLA-A2) received a multipeptide tumor antigen vaccine that was based on peptides derived from human telomerase reverse transcriptase (hTERT) and survivin, 2 “universal” tumor antigens that are often overexpressed in myeloma and may have prognostic relevance [330–332]. In this study adoptive transfer of vaccine-primed and costimulated autologous T cells generates a rapid and schedule-dependent recovery of the cellular and humoral immune system in patients with myeloma. Immune responses to a cancer vaccine occur in a substantial proportion of patients early after autotransplantation [324]. Some studies have shown high IL-6 plasma levels after ASCT [333, 334]. Condomines et al. showed that IL-7 and IL-15 plasma levels increase and peak at a median day 8 after HDM and ASCT in patients with MM [335], supporting results found in mice by Restifo and coworkers [336]. Increasing data support the idea that the early period following-lymphodepletion is propitious to promote amplification of adoptively transferred T cells and to enhance their functions. Several studies in mice and humans showed that homeostatic expansion is associated with faster and more efficient immune response and that immunization with tumor antigens during lymphopenia generates CD8 T cells with enhanced antitumor capacities [337–340]. IL-7, produced by stromal cells, is required for homeostatic expansion of naïve and memory CD4 and CD8 T cells and is critical for their survival [341]. IL-15 drives antigen-independent homeostatic memory CD8+ *αβ*T cell proliferation [341, 342]. IL-7 and IL-15 are also required for *γδ*T cell homeostatic expansion [343]. The *γ*9*δ*2T cells exert antimyeloma-specific cytotoxicity, can be expanded 100-fold with IL-2 and biphosphonate*ex vivo* [344], and are present in mobilized autografts [345]. These *γ*9*δ*2T cells could be expanded *ex vivo* and then grafted after ASCT. CD8 T cells recognizing several myeloma antigens as MUC-1 [346], cancer-testis antigens [124, 126, 127], or IgG epitopes [347], detected in peripheral blood of patients, may also be present in HSC harvests. Once stimulated *ex vivo *with antigen-pulsed DCs [348], these antimyeloma cell CD8+ T cells are able to kill myeloma cells.

## 17. Immunotherapy after Allogeneic SCT

 Allogeneic transplantation results in long-term disease-free survival for a subset of patients with MM. The unique efficacy of allogeneic transplantation is due to the graft-versus-disease effect that is mediated by alloreactive donor T cells [5, 349, 350].

 Compared with autologous transplantation, allogeneic transplantation results in lower rates of disease relapse and higher rates of molecular remission [5, 351–353]. Standard myeloablative alloSCT for myeloma is associated with a rather high treatment-related mortality. One approach to reduce transplant-related mortality is the use of reduced-intensity conditioning regimens [354] in which the primary antimyeloma cytoreductive agent is the donor lymphocytes contained in the graft or administered as part of DLI at a subsequent time point. The CR rate of allogeneic stem cell transplantation after standard myeloablative and dose-reduced conditioning ranged between 27% and 81% [354–358]. The ability of donor lymphocyte infusions (DLIs) to eradicate posttransplant disease relapse demonstrates the potency of the graft-versus-myeloma effect [37]. Because only those patients who achieved molecular remission have a high probability of long-term freedom from disease and cure [359], a higher number of CRs, especially molecular CRs, must be reached. For upgrading non-CR into CR may be used DLI as adoptive immunotherapy after allogeneic stem cell transplantation. In most reports on DLI in myeloma, DLI was given for relapse [360–362] and only a few reported on prophylactic DLI [363, 364]. Most studies till date have used relatively high T cell doses, resulting in a high rate of aGvHD up to 55%. DLI given after reduced-intensity conditioning in a dose-escalating manner resulted in less acute and chronic GVHD [363]. Ayuk et al. thus considered it important to find DLI doses that may induce a graft-versus-myeloma effect without GvHD [362]. Their data show that it is possible to achieve remission in myeloma patients who have relapsed, persistent, or progressive disease following RIC allografting with much lower T cell numbers with relatively low starting doses (1.0 10^6^ CD3^+^/kg BW for unrelated grafts and 4.7 10^6^ CD3^+^/kg BW for sibling grafts). The incidence and severity of aGvHD and cGvHD were relatively low [362]. Kroger et al. investigated the effect of DLI alone or in combination with s-thalidomide, bortezomib, and lenalidomide in patients with MM who achieved only partial remission or very good partial remission after allogeneic stem cell transplantation [365]. Fifty-nine percent of patients achieved CR, and this CR resulted in significantly improved progression-free survival at 5 years (58% versus 35%). CR by flow cytometry could be achieved in 63%, and this resulted in an even more favorable event-free survival at 5 years (74% versus 15%) [365].

## 18. Combined Donor Vaccination and Allogeneic Stem-Cell Transplantation

 A special aspect of active immunotherapy in MM is the combination of alloSCT with the induction of myeloma-specific immunity in the donor's immune system. The donor immune system is presumably naive for the patient's myeloma idiotype and therefore not tolerized or anergic. Therefore, induction of tumor-specific immunity in donors of haematopoietic stem cells for myeloma patients by idiotype immunization, followed by adoptive transfer of specific immune cells into the transplanted patient, may render allogeneic SCT from a nonspecific form of active immunotherapy into a tumor-specific therapy. In the 38C13 mouse lymphoma model, mice receiving marrow from a donor immunized with 38C13 idiotype had a statistically significant survival advantage after a lethal challenge with 38C13 lymphoma cells compared to animals transplanted with control marrow [366]. When preimmunized marrow transplantation was combined with a subsequent booster immunization, even tumor-bearing mice could evidently be cured of their disease. The protective effect was mediated by donor-derived T cells.

More recently, results from a formal clinical trial of donor idiotype immunization were reported. Five patients and their related donors received three subcutaneous vaccinations with idiotype (coupled to KLH at the 1st vaccination) and GM-CSF prior to alloSCT. All donors developed cellular and humoral anti-idiotype immune responses. After bone marrow transplantation, the three patients who survived longer than 30 days received 3 booster vaccinations with KLH-coupled idiotype and GM-CSF. Remarkably, these patients survived without evidence for disease recurrence for 5.5 to more than 8 years, and all had evidence for [367] idiotype-specific immunity after alloSCT.

One recipient suffered from chronic GvHD and was on chronic steroid therapy, while the other 2 recipients and all of the donors were medically well, without any significant complications.

 In order to avoid immunization of the healthy donor, attempts have been made to generate myeloma idiotype-specific donor immunity through *in vitro *stimulation of donor T cells with monocyte-derived, idiotype-presenting DC [368]. Implementation of this approach would permit to extend the principle of transfer of tumour-specific immunity to the vast pool of unrelated stem cell donors for alloSCT.

## 19. Conclusions

MM continues to be an incurable disease with fatal outcome for the majority of patients at advanced stages. Therefore, exploration of novel therapeutic modalities should be pursued.

Immunotherapy seems promising and may prove effective in eradicating the malignant stem cell pool that is non-proliferating and generally resistant to chemotherapy.

 Various clinical immunotherapy treatment strategies have been tested. Most of these strategies have focused on targeting idiotype-specific immunity. Idiotype-based vaccines have been shown in preclinical tests to induce or enhance idiotype-specific immunity. But clinical response is rare, occurring only in a minority of treated patients, suggesting that the effect is too weak to cause significant tumor destruction. Ideally, a tumor-specific immunotherapy should induce or expand only the beneficial immune responses mediated by CTLs (Th1 and Tc1 subsets) that have sufficient cytotoxic effects toward tumor cells but not normal cells. Further studies are warranted so to better understand the immune regulation mechanism in MM.

 TSAs continue to be identified in myeloma, and a systematic assessment and comparisons to identify the most promising candidates for clinical trials, are necessary. Vaccination with DC/tumor fusions induces antitumor immunity in a majority of the patients; however, responses are transient and not always associated with clinical benefit. One potential limiting factor is the regulatory T cells. It is necessary to develop ways to promote the expansion and increase the amount of functionally competent tumor-reactive T cells and to limit the influence of regulatory T cells in order to improve the efficacy of the DC/MM fusion vaccine. One approach is vaccination in conjunction with ASCT which facilitates vaccine response by inducing a minimal disease state and limiting the inhibitory influence of the myeloma cells. In preclinical models, stem cell transplantation results in the *in vivo* depletion of regulatory T cells, transient loss of tumor mediated tolerance, and enhanced capacity to respond to tumor vaccines [167, 168]. Exposure to lenalidomide increased cytotoxic T lymphocyte-mediated lysis autologous tumor targets indicating of the potential of cellular immunotherapy in conjunction with lenalidomide including its use as part in the DC/myeloma fusion vaccine [169].

Functionally potent DCs can be generated by stimulation with NK cells and may provide an effective source of DC-based immunotherapy in MM [174]. Modulation of inhibitory and activating NK receptor ligands on tumor cells represents a promising therapeutic approach against MM.

Perhaps the most interesting field for active immunotherapy in myeloma lies in the combination with allogeneic stem cell transplantation. This setting offers the advantage of an immune system that is unaffected by potential negative influences exerted by the tumor on the immune system. Transfer of tumor antigen-specific immunity from the donor to the myeloma patient may help to enhance the immunological efficacy of allogeneic SCT and to separate graft-versus-myeloma from graft-versus-host activity. The most crucial question to develop this concept further is whether the donor has to be immunized personally or whether efficacious, specific antitumor immunity can be induced *ex vivo* or in the transplanted patient.

MM exhibits a number of potentially valuable targets for mAb therapy that await further investigation in clinical studies. As has been the case with other cancers, mAbs, when employed as monotherapy in MM, have generally not produced impressive levels of response with respect to either response rates or extent of response in individual patients. However, preclinical results in MM cell lines and murine explant models and preliminary clinical results in patients with relapsed/refractory MM suggest that mAbs are likely to act synergistically with traditional therapies (dexamethasone), immune modulators (thalidomide, lenalidomide), and other novel therapies (such as the first-in-class proteasome inhibitor bortezomib); in addition, mAbs have shown the ability to overcome resistance to these therapies. These observations suggest that future work may be most productively directed at the rational development of multiagent therapies incorporating specific mAbs on the basis of clinical trial results and, possibly, on the identification of patient-specific MM disease factors. Indeed, many of the molecules composing the surface profile of plasma cells, such as CD38, CD138, CD162, and CD49d, are involved in the adhesive dynamics regulating the crosstalk between MM cells and the BM stromal environment. The search for new treatment strategies to improve outcomes for MM patients has led to the development of novel antibody-based therapies currently undergoing clinical evaluation.

Major progress in understanding interactions between the immune system and malignant cells will strongly augment the design of clinically more efficient study protocols in MM. Multiple different approaches are currently evaluated in clinical trials.
